# Correlation between the Severity of Metabolic Dysfunction-Associated Fatty Liver Disease and Serum Uric Acid to Serum Creatinine Ratio

**DOI:** 10.1155/2023/6928117

**Published:** 2023-01-13

**Authors:** Jing Liu, Hongye Peng, Che Wang, Yutong Wang, Rongrui Wang, Jixiang Liu, Tianhui Zhou, Shukun Yao

**Affiliations:** ^1^Graduate School of Peking Union Medical College, Beijing 100730, China; ^2^Department of Gastroenterology, China-Japan Friendship Hospital, Beijing 100029, China; ^3^Department of Infection, Guang'anmen Hospital, China Academy of Chinese Medical Sciences, Beijing, China; ^4^School of Qi Huang, Beijing University of Chinese Medicine, Beijing 100029, China; ^5^Beijing University of Chinese Medicine, Beijing 100029, China

## Abstract

**Purpose:**

As one of the most common chronic liver diseases, metabolic dysfunction-associated fatty liver disease (MAFLD) had different prognoses between mild and moderate-severe levels. Serum uric acid to serum creatinine ratio (sUA/Cr) can reflect the overall metabolic status of the body. To explore a convenient indicator to screen MAFLD and distinguish the severity of the disease, this study analyzed the correlation between sUA/Cr and the severity of MAFLD.

**Methods:**

228 participants were enrolled and divided into 2 groups, including mild MAFLD and non-MAFLD group and moderate-severe MAFLD group, based on liver/spleen computed tomography (CT) ratios. The correlations between sUA/Cr and the severity of MAFLD were analyzed by logistic and linear regression. Receiver operating characteristics (ROCs) analyzed the predictive ability of sUA/Cr for the severity of MAFLD expressed by the area under curve (AUC).

**Results:**

The level of sUA/Cr was higher in themoderate-severe MAFLD group than mild MAFLD and non-MAFLD group (6.14 ± 1.55 vs. 5.51 ± 1.19, *P* = 0.008). After adjustment for confounders, the correlation analysis showed that patients with elevated sUA/Cr had a higher risk of moderate-severe MAFLD (OR: 1.350, *P* = 0.036). A higher sUA/Cr level was associated with lower liver CT values (*β* = −0.133, *P* = 0.039) and liver/spleen CT ratio (*β* = −0.154, *P* = 0.016). sUA/Cr had the ability to discriminate the severity of MAFLD (AUC: 0.623).

**Conclusion:**

sUA/Cr was positively associated with the risk of moderate-severe MAFLD and had the predictive ability to discriminate the moderate-severe MAFLD from mild MAFLD and non-MAFLD. The sUA/Cr level was suggested to be monitored and controlled in the screening and treatment of MAFLD.

## 1. Introduction

Metabolic dysfunction-associated fatty liver disease (MAFLD), formerly named nonalcoholic fatty liver disease (NAFLD), has become one of the most common chronic liver diseases in the world [1]. It was reported to affect more than a third of the global population [2]. Mild MAFLD can be reversed through lifestyle intervention. However, moderate-severe MAFLD is predisposed to developing into steatohepatitis, cirrhosis, terminal liver failure, and hepatocellular carcinoma [3], and even associated with a higher risk of cardiovascular disease events [4]. Hence, it is significant to discriminate the severity of MAFLD and take measures timely to control the progression. As the diagnostic gold standard, the biopsy is invasive and hard to perform. Commonly, ultrasonography and computed tomography (CT) are used to classify the severity of MAFLD in clinical practice, while ultrasonography is inaccurate and CT has a risk of radiation exposure with a high cost [5]. Transient elastography (FibroScan) is a tool used to evaluate liver fatty infiltration and fibrosis by measuring liver stiffness but not available in routine physical examinations, especially in primary care [6]. Some anthropometric indexes used to diagnose MAFLD are with complex calculations and controversial diagnostic abilities [7]. Therefore, a noninvasive and convenient serum biomarker is required for the classification of the MAFLD severity.

Serum uric acid (sUA), the final product of purine metabolism, can reflect the metabolic status of human beings. The level of sUA has been proven to be associated with MAFLD in the US population [8]. In addition, renal function plays a key role in the excretion of UA via the kidney [9]. Serum creatinine (sCr), a chemical waste product of creatine, is affected by the number of muscles, meat intake, and kidney function [10]. sUA to sCr ratio (sUA/Cr) integrally represents the metabolic status excluding the influence of renal function. A few studies suggested that sUA/Cr may be associated with MAFLD [11]. The correlation between sUA/Cr and the severity of MAFLD still needs more research.

In clinical practices, since ultrasonography cannot reliably diagnose steatosis at <20%, mild MAFLD patients are frequently missed diagnosis or unreported, which results in mild MAFLD patients being blended with non-MAFLD people. In addition, mild and moderate-severe MAFLD had different prognoses. In order to be more in line with the real clinical situation, in this study, all the participants were divided into two groups, including mild MAFLD and non-MAFLD group, and moderate-severe MAFLD group, classified by the severity of MAFLD based on liver/spleen CT ratios [12]. The correlation analysis between sUA/Cr and the severity of MAFLD and the predictive ability of sUA/Cr for the severity of MAFLD were performed.

## 2. Materials and Methods

### 2.1. Study Design and Participants

This is a retrospective case-control of Chinese participants recruited at the China-Japan Friendship Hospital from January 2021 to October 2021. All participants were divided into 2 groups, one mild MAFLD and non-MAFLD group and the other moderate-severe MAFLD group.

All study protocols and materials were reviewed and approved by the Clinical Research Ethics Committee of CJFH (2018-110-K79-1). This study was conducted in accordance with the Declaration of Helsinki. All participants signed informed consent forms before enrollment.

### 2.2. Definition and Data Collection

#### 2.2.1. Inclusion Criteria

(1) Age ≥ 18 years and diagnosis of MAFLD [1]. The standards of diagnosis are based on histological, imaging, or blood biomarker evidence of hepatic steatosis combining at least one of the following three criteria:
  - Obesity or overweight: body mass index (BMI) ≥23 kg/m^2^ in Asians.
  - Lean (BMI < 23 kg/m^2^ in Asians) combining at least two metabolic risk abnormalities, including (1) waist circumference (WC) ≥90/80 cm in Asian men and women; (2) blood pressure (BP) ≥130/85 mmHg or specific drug treatment; (3) plasma triglycerides ≥150 mg/dl (≥1.70 mmol/L) or specific drug treatment; (4) plasma high-density lipoprotein cholesterol (HDL-C) <40 mg/dl (<1.0 mmol/L) for men and <50 mg/dl (<1.3 mmol/L) for women or specific drug treatment; (5) prediabetes (i.e., fasting glucose levels 100–125 mg/dl [5.6–6.9 mmol/L], or 2-hour postload glucose levels 140–199 mg/dl [7.8–11.0 mmol/L] or HbA1c 5.7%–6.4% [39–47 mmol/L]); (6) homeostasis model assessment of insulin resistance score ≥2.5; (7) plasma high-sensitivityC-reactive protein level >2 mg/L.
  - Type 2 diabetes mellitus (T_2_DM): diabetes mellitus is defined as an 8-hour fasting blood glucose >7.0 mmol/L (126 mg/dL) or a 2-hour postprandial blood glucose >11.1 mmol/L (200 mg/dL). T_2_DM is defined as diabetes mellitus due to a progressive loss of *β*-cell insulin secretion frequently on the background of insulin resistance [13].
(2) Imaging and laboratory data were completed and anthropometric indicators could be collected as required.

#### 2.2.2. Exclusion Criteria

Exclusion criteria are as follows: (a) missing important information (such as ultrasonography or CT results, height, and weight); (b) Cushing's syndrome, total parenteral nutrition, drugs (amiodarone, ammonium valproate, glucocorticoids, and methotrexate), etc., which can lead to fatty liver; (c) suffering from serious cardiovascular and cerebrovascular diseases, lung diseases, kidney diseases, and so on; (d) malignant tumors of the liver and other system; (e) pregnancy and lactation; (f) medication history of antihyperuricemic agents.

#### 2.2.3. Data Collection

The researcher administered a structured questionnaire to document specified data on demographic, health-related behaviors, previous history, and medication history. Anthropometric indices were measured by an eligible physician, including weight, height, waist circumference (WC), and blood pressure (BP). Weight and height were measured in light indoor clothing without shoes and heavy clothes, using a calibrated measuring apparatus. WC was measured using an inelastic measuring tape at midway between the lowest rib and the iliac crest. The BP was measured using an automatic electronic sphygmomanometer with the arm supported at the level of the heart. The mean readings of three replicate measurements were recorded.

Health examinations were performed in the morning after the examinees fasted overnight. Laboratory evaluation included sUA, sCr, aspartate aminotransferase (AST), alanine transaminase (ALT), gamma-glutamyl transferase (GGT), low-density lipoprotein cholesterol (LDL-C), high-density lipoprotein cholesterol (HDL-C), total cholesterol (TC), triglyceride (TG), total bilirubin (TBil), direct bilirubin (DBil), and fasting plasma glucose (FPG).

#### 2.2.4. CT Scan Method and CT Value Measurement

Images were obtained using a Philips 256-slice iCT scanner operated by an experienced CT examination technician. All subjects were scanned from the top of the diaphragm to the lower costal margin in the supine position after fasting overnight. The scan began when the subjects hold their breath at the end of the exhalation. The scan parameters were as follows: tube voltage, 120 kV; tube current, 250 mA; slice thickness and interval, 5 mm; field of view (FOV), 40 cm × 40 cm; window level, 40 Hu; window width, 400 Hu.

The measurement of CT value, which was designed referring to the research of Yu et al. [14], was as follows: three region-of-interest (ROI) in the liver and two ROIs in the spleen were selected at the level of porta hepatis to avoid blood vessels, bile ducts, and calcification. Each ROI was a circle of about 300 mm^2^. The respective means of the 3 CT values of the liver and 2 CT values of the spleen were calculated as the liver and the spleen CT value. The liver/spleen CT ratio, defined as liver CT value to spleen CT value, was calculated to reflect the degree of steatosis, which determined the severity of MAFLD.

#### 2.2.5. Definitions

Non-MAFLD was defined as people without MAFLD according to diagnostic criteria [1].

Mild MAFLD was defined as MAFLD patients with the liver/spleen CT ratio ≥0.7 but <1.0.

Moderate MAFLD was defined as MAFLD patients with the liver/spleen CT ratio ≥0.5 but <0.7.

Severe MAFLD was defined as MAFLD patients with the liver/spleen CT ratio <0.5.

Dyslipidemia was defined as TC ≥ 5.2 mmol/L, or TG ≥ 1.7 mmol/L, or LDL-C ≥ 3.4 mmol/L, or HDL-C < 1.0 mmol/L for men and <1.3 mmol/L for women [15].

Abnormal liver function was defined as AST ≥ 42 IU/L, or ALT ≥ 40 IU/L, or GGT ≥ 52 IU/L [16].

Abdominal obesity was defined as WC ≥ 90 cm for male or ≥80 cm for female [17].

### 2.3. Statistical Analysis

Data analysis was conducted using SPSS 26.0 and Medcalc 20.022 statistical software. Measurement data were expressed as mean ± standard deviation ($\bar{x}\pm s$) and analyzed by the Student's *t* test when approximately normally distributed. Continuous variables were shown as median (25th–75th percentiles) and analyzed by the Mann–Whitney *U* test as most variables were non-normally distributed. Counting data expressed as number (%) were analyzed by the chi-square test. Logistic regression was used to assess the correlation, expressed by odds ratios (ORs) with their 95% confidence intervals (CI). Linear trends were analyzed with linear regression. Areas under the curves (AUCs) with a 95% CI of receiver operating characteristic (ROC) were calculated to compare the predictive values of sUA/Cr for the level of MAFLD and determine the optimal cutoff point and the Youden index with maximum concomitant sensitivity and specificity. *P* < 0.05 was considered statistically significant.

## 3. Results

### 3.1. Comparisons of Baseline and Clinical Characteristics

A total of 228 subjects were included in this study. The demographic and clinical data of mild MAFLD and non-MAFLD subjects (*n* = 175), and moderate-severe MAFLD patients (*n* = 53) are shown in Table 1. Compared with participants in mild MAFLD and non-MAFLD group, patients in the moderate-severe MAFLD group had higher BMI, ALT, AST, sUA, sUA/Cr, percentages of male and abnormal liver function, and lower liver CT values, liver/spleen CT ratios (*P* all <0.05).

### 3.2. Logistic Regression Analysis of Assessing the Relationship between sUA/Cr and the Severity of MAFLD

**Figure 1:**
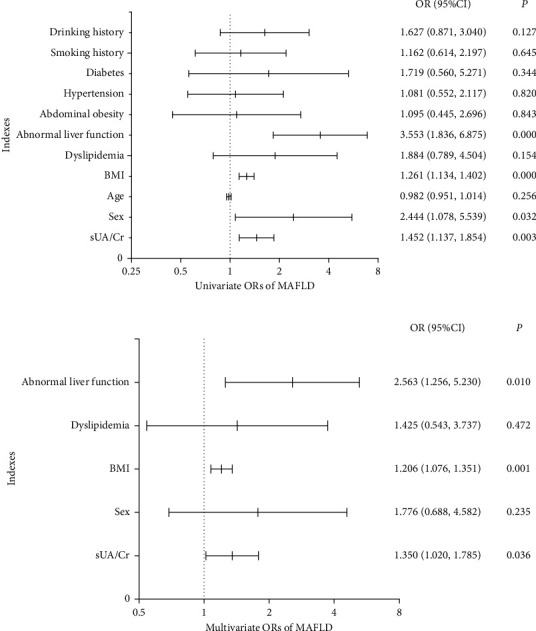
The univariate and multivariate analysis. (a) Univariate odds ratios of metabolic dysfunction-associated fatty liver disease. (b) Multivariate odds ratios of metabolic dysfunction-associated fatty liver disease. Logistic regression analysis was used to assess the relationship between serum uric acid to serum creatinine ratio and moderate-severe metabolic dysfunction-associated fatty liver disease. ORs, odds ratios; BMI, body mass index; sUA/Cr, serum uric acid to serum creatinine ratio; MAFLD, metabolic dysfunction-associated fatty liver disease.

The level of sUA/Cr in the moderate-severe MAFLD group was higher than that in the mild MAFLD and non-MAFLD group (6.14 ± 1.55 vs 5.51 ± 1.19, *P* < 0.01) (Table 1). The univariate analysis showed that sUA/Cr was positively correlated with the severity of MAFLD (OR: 1.452, 95%CI: 1.137–1.854, *P* < 0.01) (Figure 1). After adjusting for sex, BMI, dyslipidemia, and abnormal liver function, the multivariate analysis showed that sUA/Cr was still positively correlated with the severity of MAFLD (OR: 1.350, 95%CI: 1.020–1.785, *P* < 0.05) (Figure 1).

### 3.3. Linear Regression Analysis of Assessing the Relationship between sUA/Cr and Liver CT Values

The univariate analysis showed that sUA/Cr was negatively correlated with liver CT values (*β* = −0.194, *P* < 0.01) (Table 2). The multivariate analysis showed that sUA/Cr was negatively correlated with the level of MAFLD (*β* = −0.133, *P* < 0.05) after adjusting for sex, BMI, dyslipidemia, and abnormal liver function factors (Table 2).

### 3.4. Linear Regression Analysis of Assessing the Relationship between sUA/Cr and Liver/Spleen CT Ratios

The univariate analysis showed that sUA/Cr was negatively correlated with liver/spleen CT ratios (*β* = −0.204, *P* < 0.01) (Table 3). The multivariate analysis showed that sUA/Cr was negatively correlated with liver/spleen CT ratios (*β* = −0.154, *P* < 0.05) after adjusting for sex, BMI, dyslipidemia, abnormal liver function, and drinking history factors (Table 3).

### 3.5. ROC Analysis of sUA/Cr in Predicting the Severity of MAFLD

**Figure 2:**
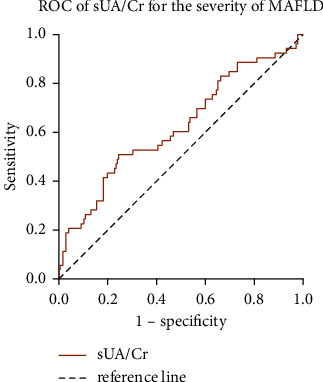
Receiver operating characteristics of serum uric acid to serum creatinine ratio for the severity of metabolic dysfunction-associated fatty liver disease. ROC, receiver operating characteristics; sUA/Cr, serum uric acid to serum creatinine ratio; MAFLD, metabolic dysfunction-associated fatty liver disease.

ROC analysis was performed to predict the ability of sUA/Cr for the severity of MAFLD. The result showed that sUA/Cr was an effective predictor in distinguishing the severity of MAFLD. The area of ROC was 0.623 (95% CI: 0.557–0.687, *P* < 0.01) with a cutoff value of 6.268, sensitivity and specificity values of 50.94% and 75.43%, respectively, and a Youden index score of 0.264 (Figure 2).

## 4. Discussion

Given the major significance of discriminating the severity of MAFLD for prognosis and the disadvantages of commonly recommended examination methods, this study analyzed the correlation between sUA/Cr and the severity of MAFLD.

MAFLD is the liver manifestation of abnormal metabolism. As an end product of purine metabolism, UA is mainly synthesized from adenine- and guanine-based purines by the enzyme xanthine oxidase [18], which plays a vital role in lipid metabolism [19]. Even though the sUA level was within the normal range, its elevated level was significantly associated with hyperlipemia and atherosis [20]. Ali et al. demonstrated that sUA was positively correlated with TG, TC, and LDL-C, and negatively with HDL-C [21]. A retrospective cohort study in the Japanese population revealed that in addition to the baseline sUA level, a higher sUA changing trajectory was positively associated with fatty liver disease (FLD) risk independently [22]. A meta-analysis conducted by Zhou and colleagues that included 9 observational studies also showed that the risk of FLD with a high sUA level was 1.92 times higher than that in patients with a low sUA level [23]. The association between sUA and MAFLD may be due to the fact that sUA could interact with oxidants and induce the production of free radicals and oxidative stress [24], which are key factors in the development of FLD [25]. Therefore, sUA, as a pro-oxidant, may have a direct effect on FLD. Besides, sUA accelerated chronic inflammatory processes by stimulating the production of proinflammatory mediators. In addition, insulin resistance (IR) was known as a risk factor for the development and progression of hepatic steatosis and metabolic syndrome (MetS) [26].

The kidney could regulate hyperuricemia by modulating urinary uric acid excretion. A lower glomerular filtration rate elevates the level of sUA, and Cr is commonly used as an indicator of renal function. Compared with sUA, sUA/Cr is more reasonable to accurately reflect the endogenous UA level. Kawamoto et al. indicated that sUA/Cr was the independent predictor of MetS [27]. In this study, both sUA levels and sUA/Cr were significantly higher in the moderate-severe MAFLD group than that in the mild MAFLD and non-MAFLD groups. The severity of MAFLD was positively associated with increased sUA/Cr, even after adjustment for sex, BMI, dyslipidemia, and abnormal liver function. In line with our results, Han and Lee found MAFLD was related to sUA/Cr, and the amount of alcohol consumption and smoking influenced the association [28]. Similarly, Liu et al. observed that the strength of association between sUA/Cr and MetS showed a linear dose-response relationship [29]. Ma et al. demonstrated that sUA/Cr was an independent risk factor of NAFLD in individuals with normal sUA levels and had a direct effect on NAFLD by mediation analysis [11].

In addition, this study found that sUA/Cr had the predictive ability to discriminate the severity of MAFLD. When sUA/Cr ≥ 6.268, the risk of moderate-severe MAFLD was significantly increased. In another Chinese study, sUA/Cr had good sensitivity and specificity in predicting the risk of FLD in subjects. When SUA/Cr ≥ 4.66, the risk of FLD was significantly increased [30].

### 4.1. Strengths

There are some advantages in this study. sUA/Cr was introduced as a relatively new biological indicator and revealed a significant association between sUA/Cr and the severity of MAFLD in a fast, convenient, and noninvasive way, thus providing a more accurate prediction for the classification of MAFLD. The level of sUA/Cr can help clinical doctors rapidly distinguish the severity of MAFLD and guide them to develop more personalized intervention strategies for different patients, especially for moderate-severe patients to give proper treatment as early as possible to improve the prognosis. It is necessary for patients in poor and rural areas where inspection conditions are limited.

### 4.2. Limitations

Firstly, this study was a cross-sectional study and could not verify the causality between elevated sUA/Cr level and the severity of MAFLD and which one is the causal factor or the consequence. Further exploration of the causal effect of sUA/Cr on MAFLD will be needed. Secondly, MAFLD was diagnosed by CT and blood tests without biopsy, which is considered as the gold standard. However, CT is still a noninvasive and efficient tool for the diagnosis of steatosis. Finally, the objects in this study were from the Chinese population, and the findings may not be generalizable to other countries.

## 5. Conclusion

The risk of moderate-severe MAFLD was positively associated with increased sUA/Cr, even after adjustment for sex, BMI, dyslipidemia, and abnormal liver function. The level of sUA/Cr had the ability to discriminate the moderate-severe MAFLD from mild MAFLD and non-MAFLD. Although the UA is not included in the diagnostic standards of MAFLD, this study suggested that the sUA/Cr level should be monitored and controlled in the screening and treatment of MAFLD, which contributes to preventing the deterioration of the disease.

## Figures and Tables

**Table 1:** Characteristics of study participants (*n* = 228).

	Mild MAFLD and non-MAFLD (*n* = 175)	Moderate-severeMAFLD (*n* = 53)	*P*
Male [*n* (%)]	122 (69.7%)	45 (84.9%)	0.029
Female [*n* (%)]	53 (30.3%)	8 (15.1%)	
Age (years)	47.0 (41.0–55.0)	48.0 (36.5–53.0)	0.266
BMI (kg/m^2^)	26.92 ± 3.02	29.39 ± 3.74	<0.001
Liver CT value	50.39 ± 7.36	30.63 ± 6.73	<0.001
Liver/spleen CT ratio	0.933 (0.847–1.078)	0.609 (0.488–0.671)	<0.001
ALT (mmol/L)	30.0 (21.0–41.0)	45.0 (34.0–69.5)	<0.001
AST (mmol/L)	22.0 (18.0–26.0)	26.0 (21.5–35.5)	<0.001
TBil (*μ*·mol/L)	12.43 (9.20–16.78)	12.180 (9.965–17.165)	0.623
DBil (*μ*·mol/L)	1.94 (1.56–2.54)	2.250 (1.690–2.845)	0.103
sUA (*μ*·mol/L)	354.47 ± 79.48	412.77 ± 75.12	<0.001
sCr (*μ*·mol/L)	65.80 ± 14.62	69.16 ± 11.81	0.091
sUA/Cr	5.51 ± 1.19	6.14 ± 1.55	0.008
TG (mmol/L)	1.84 (1.32–2.83)	2.050 (1.485–2.570)	0.333
FPG (mmol/L)	5.57 (5.17–6.04)	5.690 (5.340–6.665)	0.075
Dyslipidemia (*n* (%))	136 (77.7%)	46 (86.8%)	0.149
Abnormal liver function (*n* (%))	69 (39.4%)	37 (69.8%)	<0.001
Hypertension (*n* (%))	50 (28.6%)	16 (30.2%)	0.820
Diabetes (*n* (%))	10 (5.7%)	5 (9.4%)	0.339
Abdominal obesity (*n* (%))	150 (85.7%)	46 (86.8%)	0.843
Smoking history (*n* (%))	60 (34.3%)	20 (37.7%)	0.645
Drinking history (*n* (%))	59 (33.7%)	24 (45.3%)	0.125

Data were expressed as mean ± standard deviation and analyzed by Student's *t*-test when approximately normally distributed or otherwise expressed as median (25th–75th percentiles) and analyzed by the Mann–Whitney *U* test for continuous variables or expressed as number (percentage) and analyzed by chi-square test for categorical variables. MAFLD, metabolic-associated fatty liver disease; BMI, body mass index; CT, computed tomography; ALT, alanine transaminase; AST, aspartate aminotransferase; TBil, total bilirubin; DBil, direct bilirubin; sUA, serum uric acid; sCr, serum creatinine; sUA/Cr, serum uric acid to serum creatinine ratio; TG, triglyceride; TC, total cholesterol; FPG, fasting plasma glucose.

**Table 2:** Univariate and multivariate linear regression analyses of liver computed tomography value.

Analysis method	Indicators	Unstandardized coefficients		Standardized coefficients	*t*	*P*
		*B*	Std. error	*β*		
*Univariate analysis*						
sUA/Cr		−1.634	0.551	−0.194	−2.967	0.003
Sex		−4.643	1.626	−0.187	−2.856	0.005
Age		0.085	0.076	0.074	1.120	0.264
BMI		−0.985	0.208	−0.300	−4.727	<0.001
Dyslipidemia		−6.126	1.780	−0.223	−3.442	0.001
Abnormal liver function		−7.444	1.383	−0.337	−5.383	<0.001
Abdominal obesity		−3.025	2.100	−0.095	−1.441	0.151
Hypertension		−0.976	1.614	−0.040	−0.604	0.546
Diabetes		−2.098	2.952	−0.047	−0.711	0.478
Smoking history		−2.358	1.527	−0.102	−1.544	0.124
Drinking history		−3.437	1.505	−0.150	−2.283	0.023

*Multivariate analysis*						
sUA/Cr		−1.119	0.538	−0.133	−2.080	0.039
Sex		−1.779	1.720	−0.072	−1.034	0.302
BMI		−0.621	0.210	−0.189	−2.952	0.004
Dyslipidemia		−4.320	1.681	−0.157	−2.570	0.011
Abnormal liver function		−5.317	1.399	−0.241	−3.801	<0.001
Drinking history		−0.556	1.510	−0.024	−0.369	0.713

sUA/Cr, serum uric acid to serum creatinine ratio; BMI, body mass index.

**Table 3:** Univariate and multivariate linear regression analyses of liver/spleen computed tomography ratio.

Analysis method	Indicators	Unstandardized coefficients		Standardized coefficients	*t*	*P*
		*B*	Std. error	*β*		
*Univariate analysis*						
sUA/Cr		−0.033	0.011	−0.204	−3.126	0.002
Sex		−0.112	0.031	−0.230	−3.555	<0.001
Age		0.002	0.001	0.099	1.502	0.135
BMI		−0.020	0.004	−0.308	−4.863	<0.001
Dyslipidemia		−0.114	0.035	−0.214	−3.292	0.001
Abnormal liver function		−0.147	0.027	−0.342	−5.472	<0.001
Abdominal obesity		−0.064	0.041	−0.104	−1.576	0.116
Hypertension		−0.028	0.031	−0.059	−0.881	0.379
Diabetes		−0.049	0.057	−0.057	−0.853	0.395
Smoking history		−0.050	0.030	−0.112	−1.693	0.092
Drinking history		−0.067	0.029	−0.151	−2.289	0.023

*Multivariate analysis*						
sUA/Cr		−0.025	0.010	−0.154	−2.439	0.016
Sex		−0.064	0.033	−0.131	−1.914	0.057
BMI		−0.012	0.004	−0.184	−2.892	0.004
Dyslipidemia		−0.075	0.032	−0.141	−2.319	0.021
Abnormal liver function		−0.101	0.027	−0.236	−3.753	<0.001
Drinking history		−0.003	0.029	−0.008	−0.117	0.907

sUA/Cr, serum uric acid to serum creatinine ratio; BMI, body mass index.

## Data Availability

All data relevant to the study are included in the article. Data can be provided upon request to credible investigators on verification for patient confidentiality.

## References

[1] Eslam M., Newsome P. N., Sarin S. K. (2020). A new definition for metabolic dysfunction-associated fatty liver disease: an international expert consensus statement. *Journal of Hepatology*.

[2] Chan K. E., Koh T. J. L., Tang A. S. P. (2022). Global prevalence and clinical characteristics of metabolic-associated fatty liver disease: a meta-analysis and systematic review of 10 739 607 individuals. *Journal of Clinical Endocrinology and Metabolism*.

[3] Serfaty L., Lemoine M. (2008). Definition and natural history of metabolic steatosis: clinical aspects of NAFLD, NASH and cirrhosis. *Diabetes and Metabolism*.

[4] Lee H., Lee Y. H., Kim S. U., Kim H. C. (2021). Metabolic dysfunction-associated fatty liver disease and incident cardiovascular disease risk: a nationwide cohort study. *Clinical Gastroenterology and Hepatology*.

[5] Idilman I. S., Ozdeniz I., Karcaaltincaba M. (2016). Hepatic steatosis: etiology, patterns, and quantification. *Seminars in Ultrasound, CT and MRI*.

[6] Foucher J., Chanteloup E., Vergniol J., Castera L., Bail B. L., Bertet J. (2006). Diagnosis of cirrhosis by transient elastography (FibroScan): a prospective study. *Gut*.

[7] Liu J., Duan S., Wang C. (2022). Optimum non-invasive predictive indicators for metabolic dysfunction-associated fatty liver disease and its subgroups in the Chinese population: a retrospective case-control study. *Frontiers in Endocrinology*.

[8] Shih M. H., Lazo M., Liu S. H., Bonekamp S., Hernaez R., Clark J. M. (2015). Association between serum uric acid and nonalcoholic fatty liver disease in the US population. *Journal of the Formosan Medical Association*.

[9] Ndrepepa G. (2018). Uric acid and cardiovascular disease. *Clinica Chimica Acta*.

[10] Perrone R. D., Madias N. E., Levey A. S. (1992). Serum creatinine as an index of renal function: new insights into old concepts. *Clinical Chemistry*.

[11] Ma C., Liu Y., He S. (2020). C-peptide: a mediator of the association between serum uric acid to creatinine ratio and non-alcoholic fatty liver disease in a Chinese population with normal serum uric acid levels. *Frontiers in Endocrinology*.

[12] Association F. LaA. L. D. S. GoC. L. D. (2003). Diagnostic criteria of nonalcoholic fatty liver disease. *Chinese Journal of Hepatology*.

[13] American Diabetes Association (2017). 2. Classification and diagnosis of diabetes. *Diabetes Care*.

[14] Yu A. H., Duan-Mu Y. Y., Zhang Y. (2018). Correlation between non-alcoholic fatty liver disease and visceral adipose tissue in non-obese Chinese adults: a CT evaluation. *Korean Journal of Radiology*.

[15] Rader D. J., Hoeg J. M., Brewer H. B. (1994). Quantitation of plasma apolipoproteins in the primary and secondary prevention of coronary artery disease. *Annals of Internal Medicine*.

[16] Cheng T. J., Huang M. L., You N. C., Du C. L., Chau T. T. (1999). Abnormal liver function in workers exposed to low levels of ethylene dichloride and vinyl chloride monomer. *Journal of Occupational and Environmental Medicine*.

[17] Dhawan D., Sharma S. (2020). Abdominal obesity, adipokines and non-communicable diseases. *The Journal of Steroid Biochemistry and Molecular Biology*.

[18] Yao J. K., Dougherty G. G., Reddy R. D. (2010). Homeostatic imbalance of purine catabolism in first-episodeneuroleptic-naïve patients with schizophrenia. *PLoS One*.

[19] Chen M. Y., Zhao C. C., Li T. T. (2017). Serum uric acid levels are associated with obesity but not cardio-cerebrovascular events in Chinese inpatients with type 2 diabetes. *Scientific Reports*.

[20] Lima W. G., Martins-Santos M. E. S., Chaves V. E. (2015). Uric acid as a modulator of glucose and lipid metabolism. *Biochimie*.

[21] Ali N., Rahman S., Islam S. (2019). The relationship between serum uric acid and lipid profile in Bangladeshi adults. *BMC Cardiovascular Disorders*.

[22] Jensen T., Niwa K., Hisatome I. (2018). Increased serum uric acid over five years is a risk factor for developing fatty liver. *Scientific Reports*.

[23] Zhou Y., Wei F., Fan Y. (2016). High serum uric acid and risk of nonalcoholic fatty liver disease: a systematic review and meta-analysis. *Clinical Biochemistry*.

[24] Sautin Y. Y., Imaram W., Kim K. M., Angerhofer K. M., Henderson A., Johnson J., Miyata T., Eckardt K. U., Nangaku M. (2011). *Uric Acid and Oxidative Stress*.

[25] Spahis S., Delvin E., Borys J. M., Levy E. (2017). Oxidative stress as a critical factor in nonalcoholic fatty liver disease pathogenesis. *Antioxidants and Redox Signaling*.

[26] Li C., Hsieh M. C., Chang S. J. (2013). Metabolic syndrome, diabetes, and hyperuricemia. *Current Opinion in Rheumatology*.

[27] Kawamoto R., Ninomiya D., Akase T. (2019). Serum uric acid to creatinine ratio independently predicts incident metabolic syndrome among community-dwelling persons. *Metabolic Syndrome and Related Disorders*.

[28] Han A. L., Lee H. K. (2022). Association of the metabolic dysfunction-associated fatty liver disease with serum uric acid-to-creatinine ratio. *Metabolic Syndrome and Related Disorders*.

[29] Liu S., Song J., Peng J., Tang Z., Zhang J., Zhang L. (2020). Association of serum uric acid/creatinine ratio and metabolic syndrome in euthyroid population. *Journal of Hygiene Research*.

[30] Xie M., Zheng J., Chen S. (2022). Association of serum uric acid/creatinine ratio with nonalcholic fatty liver disease in adults. *Zhejiang Journal of Integrated Traditional Chinese and Western Medicine*.

